# Cigarette smoking in childhood and risk of all-cause and cause-specific mortality in adulthood

**DOI:** 10.3389/fpubh.2023.1051597

**Published:** 2023-07-06

**Authors:** Xue Liu, Jiahong Sun, Min Zhao, Pascal Bovet, Bo Xi

**Affiliations:** ^1^Department of Epidemiology, School of Public Health, Qilu Hospital, Cheeloo College of Medicine, Shandong University, Jinan, China; ^2^Department of Toxicology and Nutrition, School of Public Health, Cheeloo College of Medicine, Shandong University, Jinan, China; ^3^Center for Primary Care and Public Health (Unisanté), University of Lausanne, Lausanne, Switzerland

**Keywords:** childhood smoking, mortality, all-cause and cause-specific, smoking initiation, smoking cessation

## Abstract

**Background:**

This study was aimed to examine the association between cigarette smoking in childhood and mortality in adulthood, and the impact of non-smoking duration among smokers who subsequently quit smoking.

**Methods:**

We used data from 472,887 adults aged 18–85 years examined once in the US National Health Interview Survey in 1997–2014, which was linked to mortality data from the National Death Index up to 31 December 2015. Cigarette smoking status in childhood (age 6 to 17 years) and adulthood (age 18 to 85 years) was self-reported using a standard questionnaire at the time of participation in the survey. The vital status of participants due all-causes, cardiovascular disease (CVD), cancer and chronic lower respiratory diseases was obtained using mortality data from the National Death Index.

**Results:**

During the mean follow-up of 8.75 years, compared with never smoking in childhood and adulthood, the risk of all-cause mortality among current adult smokers decreased slightly according to increasing age at smoking initiation: hazard ratios (HRs; 95% confidence intervals, CIs) were 2.54 (2.24–2.88) at age of 6–9 years, 2.44 (2.31–2.57) at age of 10–14 years, and 2.21 (2.12–2.31) at age of 15–17 years. Smoking cessation before the age of 30 years was not associated with increased risk of all-cause and cause-specific mortality (all *p* > 0.05) compared to never smoking.

**Conclusion:**

Mortality risk was higher in individuals who started smoking at an earlier age in childhood. Inversely, smoking cessation before the age of 30 years was not associated with an increased risk of mortality compared to never smoking.

## Introduction

Despite the declining prevalence of cigarette smoking among US adults from 20.9% in 2005 to 16.8% in 2014 (1), cigarette use remains the leading modifiable cause of non-communicable chronic diseases, particularly cardiovascular disease (CVD), cancer, and premature mortality in the US (1), as well as in other countries (2). Reducing the burden of the smoking-related morbidity and mortality requires comprehensive public health measures to reduce the demand for cigarettes, with a particular focus to prevent smoking initiation at an early age, and clinical measures to promote smoking cessation among smokers (3, 4).

Few studies have examined the effect of age at smoking initiation and cessation with all-cause and cause-specific mortality. The China Kadoorie Biobank including 0.5 million Chinese adults aged 35–74 years suggested that adult smokers who started smoking before the age of 20 years had a higher risk of all-cause and cause-specific mortality compared to adult smokers who started to smoke after the age of 20 years (5). A recent cohort study including 118,840 adults aged 30–69 years from Cuba showed that adult smokers who had already smoked cigarettes in childhood (5–19 years) had a higher risk of premature death compared with adult smokers who started smoking after the age of 20 years (6). In addition, this cohort suggested that smoking cessation before the age of 40 years was not associated with excess mortality attributable to smoking (6). Although the studies highlight the increased mortality risk due to early smoking initiation among adult smokers (7), we are not aware of studies that have examined the association between change in smoking status from childhood to adulthood and mortality risk in adulthood. In addition, although few studies have shown that quitting smoking before the age of 40 years could avoid much of the excess all-cause deaths attributable to smoking (6), it is unclear whether this age limit of 40 years for smoking cessation similarly applies to mortality from different smoking-related diseases (e.g., CVD, cancer or chronic lower respiratory diseases).

Therefore, we examined the associations of cigarette smoking in childhood (age 6 to 17 years), change in cigarette smoking status from childhood to adulthood (age 18 to 85 years), and age of smoking cessation in adulthood, with all-cause and cause-specific (cancer, CVD, or chronic lower respiratory diseases) mortality in participants aged 18 to 85 years at the time of their participation in the U.S. National Health Interview Survey (NHIS) in 1997–2014 and for whom cause-specific mortality status was linked with the U.S national vital statistics up to 2015.

## Methods

### Study population

The NHIS is a nationally representative household survey in the United States using a face-to-face interview to collect information on health at the time of the survey and retrospective information at the time childhood. Methods and descriptions of the NHIS have been widely reported elsewhere (8, 9). Briefly, the NHIS uses a geographically clustered sampling design to randomly select households, and one adult from each selected household was submitted to a detailed face-to-face interview on health. The NHIS is conducted by the National Center for Health Statistics (NCHS) of the Centers for Disease Control and Prevention (CDC) since 1957. The NHIS anonymized data are publicly available and ethical review by the corresponding author’s ethics committee is not requested.

For this study, we used NHIS data collected in 1997–2014 with linkage to mortality data up to 31 December 2015. We used NHIS data collected after 1996 due to a major revision of the NHIS questionnaire in 1997. Data were available for a total of 529,363 participants aged 18–85 years. No participant attended a NHIS more than one time. Among them, 56,476 were excluded based on the study design and objectives in this study owing to pregnancy (*n* = 6,364); missing data on current smoking status (*n* = 3,299); missing data on age at smoking initiation (*n* = 8,736); missing data on age at smoking cessation (*n* = 595); missing data on potential covariates used for analysis in this study (demographic variables, lifestyle factors, and co-morbidities; *n* = 37,482), resulting in a final analytic sample of 472,887 participants.

### Mortality outcomes

Mortality outcomes in this study were obtained from the National Death Index (NDI) records. NCHS created the Linked Mortality File (LMF) with identifiable variable using a probabilistic matching method described elsewhere (10), with a combination of social security number (SSN), name, date of birth, and sex, which had a good success rate of 96% (91%–98%) (11). The underlying cause of deaths were coded along the International Classification of Diseases 10th Revision (ICD-10). We considered (1) all-cause mortality, (2) cancer mortality (codes C00 to C97), (3) CVD mortality (codes I00 to I09, I11, I13, I20 to I51, and I60 to I69), and (4) chronic lower respiratory diseases (codes J40-J47). Death from any underlying leading cause was defined as an all-cause death. If the underlying leading cause of death is from a cancer, CVD, or chronic lower respiratory disease, death was defined as a cause-specific death in this study. Participants to the surveys who were not identified in the NDI records were considered to be alive. The follow-up duration was quantified as the time interval between when a participant had attended a NHIS survey in 1997–2014 and the date of the linkage with the NDI records (on 31 December 2015). The accuracy of all-cause and cause-specific mortality recorded in the NDI database has been validated previously (12).

### Cigarette smoking

Information on cigarette smoking in childhood (i.e., recall) and in adulthood (i.e., current status) was based on self-reported information through a standard questionnaire at the time a participant attended the NHIS. Cigarette smoking status in adulthood was defined as never, former and current smoking using the following questions: (Q1) “Have you smoked at least 100 cigarettes in your ENTIRE LIFE?” (Yes vs. No); (Q2) “Do you NOW smoke cigarettes?” (Yes vs. No). According the responses to these 2 questions, adults were categorized into never smoker (“No” to Q1), former smoker (“Yes” to Q1, “No” to Q2), and current smoker (“Yes” to Q2). Adults who ever smoked at least 100 cigarettes during their entire life were asked a question on age at smoking uptake (“How old were you when you FIRST started to smoke fairly regularly?).” We categorized all former and current adult smokers into 4 categories: smoking initiation at age 6–10, 10–14, 15–17, and ≥18 years. Cigarette smoking status in childhood and adulthood was based on the age at smoking initiation and age at smoking cessation, categorized into 4 categories: never smoker during childhood and adulthood (never smoker group); smoker during childhood but has quit in adulthood (cessation group); never smoker during childhood but current smoker during adulthood (incident smoker group); smoker during childhood and current smoker during adulthood (persistent smoker group). Time since quitting smoking (in years) was categorized into <5, 5–9, 10–19, 20–29, and ≥30 years. Age of smoking cessation was calculated as the adult’s current age at the NHIS data collection minus the duration (years) since quitting smoking that was subsequently divided into four categories (<30, 30–39, 40–49, and ≥50 years).

### Study covariates

Personal information on socio-demographic characteristics, lifestyle factors and chronic conditions was self-reported using a standard questionnaire. Socio-demographic characteristics included age, sex, race/ethnicity (White and not Hispanic, Black, Hispanic, and others), education (lower than high school, high school, and higher than high school), marital status (married; divorced/separated/widowed, and never married). Body mass index (BMI, weight divided by height squared, kg/m^2^) was categorized into <25.0, 25.0–29.9, ≥30.0 kg/m^2^. Behavioral factors included drinking status [lifetime abstainer, former drinker, current light to moderate drinker (1–7 drinks/week for women and 1–14 drinks/week for men), and current heavy drinker (>1 drinks/day for women and >2 drinks/day for men)] (13), physical activity [PA, whether or not meet the Physical Activity Guidelines for Americans (at least 75 min of vigorous PA or 150 min of moderate PA in 1 week or an equivalent of the combination)] (14). Self-reported physician-diagnosed chronic conditions included hypertension, heart disease, stroke, cancer, and diabetes.

### Statistical analysis

Statistics about basic characteristics were shown as percentages for categorical variables. Study participants were divided into nine categories based on cigarette smoking status and age at smoking initiation (never smokers, smoking initiation at 6–9, 10–14, 15–17, ≥18 years for both current and former smokers).

Cox proportional hazards regression models (data meeting the assumption) were performed to estimate the hazard ratios (HRs) and 95% confidence intervals (CIs) of cigarette smoking status with all-cause and cause-specific mortality with adjustment for potential confounders. Three multivariable adjustment models were processed as follows: Model 1: adjusted for age, sex, race/ethnicity. Model 2: Model 1 plus education, and marital status, BMI, drinking, and PA. Model 3: Model 2 plus physician-diagnosed diseases. Additionally, two sensitivity analyses were also performed to assess the stability of our results: (1) a sensitivity analysis under the exclusion of those who died within the first 2 years of follow up to avoid the deaths which were not caused by smoking within a short duration; (2) a sensitivity analysis under the exclusion of those with any chronic diseases identified through the NHIS survey to avoid possible reverse causation associations.

All statistical analyses were performed using SAS version 9.3 (SAS Institute Inc., Cary, North Carolina) in consideration of sampling weights, strata and primary sampling units in the NHIS to obtain nationally representative estimates. Two-sided *p* < 0.05 was considered statistically significant.

## Results

Overall, 472,887 participants were included in the main analyses, including 271,884 (57.5%) never smokers, 101,089 (21.4%) former smokers and 99,914 (21.1%) current smokers. There were significant differences in sociodemographic, lifestyle factors, and chronic conditions across cigarette smoking status at baseline (*p* < 0.0001, Supplementary Table 1). Compared with never smokers, the former and current smokers more likely to be older, male, white, have low education (high school education or less), divorced/separated/widowed, overweight or obese, former or current drinkers, have not met recommended physical activity levels, and have more physician-diagnosed chronic conditions. In addition, both former and current smokers with smoking initiation before the age of 10 years were more likely to be older, male, have low education (<high school education), divorced/separated/widowed, obese, former drinkers, have not met recommended physical activity levels, and have more physician-diagnosed chronic conditions (Supplementary Table 1).

### Age at smoking initiation and the risk of all-cause and cause-specific mortality

During the mean follow up of 8.75 years, death was recorded in 58,096 participants, including 25,784 among never smokers, 19,468 among former smokers and 13,249 current smokers. Among all deaths, 14,091 died from cancer, 13,198 from CVD and 3,130 from chronic lower respiratory tract diseases.

**Figure 1:**
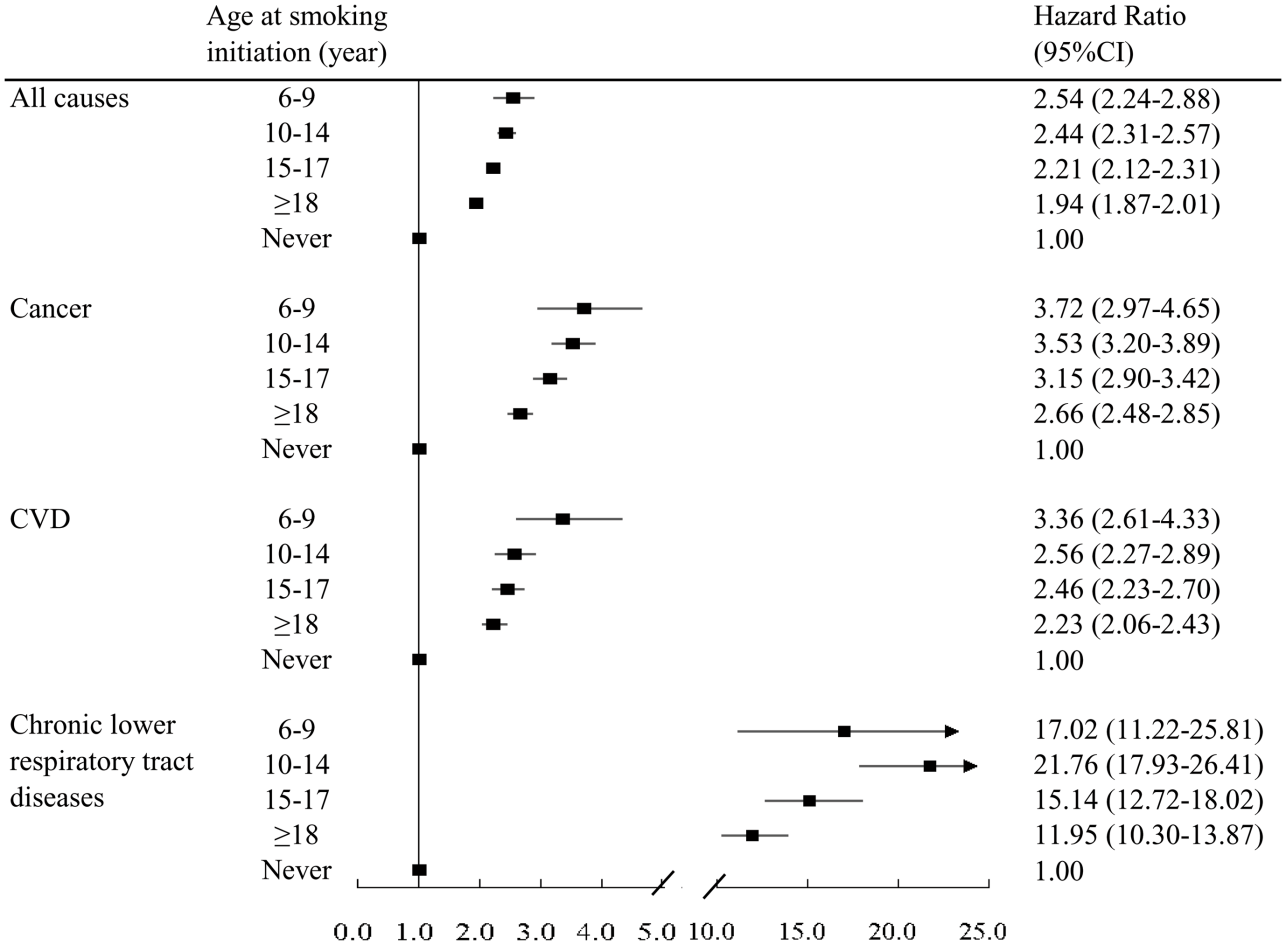
Association between age at smoking initiation and all-cause and cause-specific mortality among participants who smoked during childhood and adulthood (persistent smokers). Model was adjusted for age, gender, race/ethnicity, education, marital status, body mass index, alcohol intake, physical activity, and chronic conditions.

Using cox proportional hazards regression models, we found that the risk of mortality in adulthood from all causes and from major causes was significantly higher among former and current smokers irrespective of smoking initiation age (Table 1). In the fully adjusted model (model 3), relative to never smokers, the HRs (95% CIs) of all-cause mortality were 1.38 (1.21–1.56), 1.41 (1.35–1.48), 1.30 (1.25–1.34), and 1.25 (1.22–1.28) for former smokers who started smoking at 6–9, 10–14, 15–17, and ≥18 years, respectively, and were 2.54 (2.24–2.88), 2.44 (2.31–2.57), 2.21 (2.12–2.31), and 1.94 (1.87–2.01) for current smokers who started smoking at 6–9, 10–14, 15–17, and ≥18 years, respectively (Table 1; Figure 1). Similar findings were observed for considered specific causes of deaths (Table 1; Figure 1). Excess mortality associated with smoking was highest for chronic lower respiratory tract diseases-specific mortality, followed by cancer-specific mortality and CVD-specific mortality.

**Table 1:** Association between age at smoking initiation and all-cause and cause-specific mortality.

Outcome	Never	Age at smoking initiation for participants who smoked during childhood but had quit smoking during adulthood				Age at smoking initiation for participants who smoked during childhood and were currently smoking during adulthood			
		6–9	10–14	15–17	≥18	6–9	10–14	15–17	≥18
***N***	271,884	1,460	16,024	34,005	49,600	1,650	17,979	33,907	46,378
**All causes**									
No of deaths:	25,784	449	3,140	5,953	9,926	398	2,628	4,148	6,075
Model 1	1.00	1.71 (1.52–1.93)	1.52 (1.46–1.59)	1.30 (1.25–1.34)	1.19 (1.15–1.22)	3.31 (2.92–3.74)	2.91 (2.77–3.06)	2.46 (2.36–2.57)	2.00 (1.92–2.07)
Model 2	1.00	1.60 (1.41–1.81)	1.56 (1.49–1.63)	1.39 (1.34–1.44)	1.33 (1.29–1.36)	2.82 (2.50–3.19)	2.62 (2.49–2.76)	2.30 (2.20–2.40)	1.97 (1.90–2.05)
Model 3	1.00	1.38 (1.21–1.56)	1.41 (1.35–1.48)	1.30 (1.25–1.34)	1.25 (1.22–1.28)	2.54 (2.24–2.88)	2.44 (2.31–2.57)	2.21 (2.12–2.31)	1.94 (1.87–2.01)
**Cancer**									
No of deaths:	5,248	112	845	1,595	2,298	119	816	1,288	1770
Model 1	1.00	2.28 (1.82–2.86)	1.99 (1.82–2.17)	1.68 (1.57–1.79)	1.41 (1.33–1.50)	4.73 (3.83–5.85)	4.14 (3.76–4.56)	3.49 (3.22–3.77)	2.73 (2.55–2.92)
Model 2	1.00	2.13 (1.70–2.68)	2.00 (1.83–2.19)	1.74 (1.63–1.86)	1.53 (1.44–1.63)	4.16 (3.36–5.15)	3.79 (3.44–4.19)	3.28 (3.02–3.55)	2.70 (2.52–2.89)
Model 3	1.00	1.77 (1.40–2.25)	1.84 (1.68–2.02)	1.62 (1.52–1.74)	1.44 (1.36–1.53)	3.72 (2.97–4.65)	3.53 (3.20–3.89)	3.15 (2.90–3.42)	2.66 (2.48–2.85)
**CVD**									
No of deaths:	6,136	104	700	1,302	2,330	112	488	779	1,247
Model 1	1.00	1.65 (1.25–2.20)	1.49 (1.36–1.63)	1.17 (1.09–1.26)	1.15 (1.09–1.21)	4.76 (3.71–6.10)	3.26 (2.90–3.66)	2.81 (2.55–3.09)	2.33 (2.17–2.50)
Model 2	1.00	1.50 (1.12–2.00)	1.53 (1.40–1.68)	1.28 (1.19–1.38)	1.34 (1.26–1.42)	3.92 (3.06–5.02)	2.88 (2.55–3.25)	2.61 (2.37–2.88)	2.32 (2.15–2.51)
Model 3	1.00	1.22 (0.90–1.65)	1.33 (1.21–1.46)	1.16 (1.08–1.25)	1.22 (1.15–1.30)	3.36 (2.61–4.33)	2.56 (2.27–2.89)	2.46 (2.23–2.70)	2.23 (2.06–2.43)
**Chronic lower respiratory tract diseases**									
No of deaths:	494	43	277	466	695	37	254	337	527
Model 1	1.00	11.63 (7.83–17.28)	9.38 (7.83–11.23)	6.50 (5.48–7.70)	4.90 (4.28–5.63)	27.82 (18.55–41.73)	29.78 (24.65–35.99)	18.87 (15.92–22.36)	13.58 (11.76–15.69)
Model 2	1.00	10.63 (7.00–16.15)	9.94 (8.29–11.92)	7.29 (6.11–8.69)	5.94 (5.15–6.85)	20.34 (13.44–30.76)	23.55 (19.41–28.59)	15.75 (13.24–18.73)	12.27 (10.60–14.21)
Model 3	1.00	9.04 (5.88–13.91)	9.05 (7.55–10.86)	6.79 (5.71–8.09)	5.56 (4.82–6.41)	17.02 (11.22–25.81)	21.76 (17.93–26.41)	15.14 (12.72–18.02)	11.95 (10.30–13.87)

Results are expressed as hazard ratios and 95% confidence intervals. Model 1: Adjusted for sex, age, and race/ethnicity. Model 2: Model 1 + education, marital status, body mass index, alcohol intake, and physical activity. Model 3: Model 2 + chronic conditions.

### Changes in cigarette smoking status from childhood to adulthood and risk of all-cause and cause-specific mortality

Among the 421,647 participants, never smoker group accounted for 64.5% (*n* = 271,884), cessation group 11.8% (*n* = 49,849), incident smoker group 11.0% (*n* = 46,378), and persistent smoker group 12.7% (*n* = 53,536).

In the fully adjusted model (Supplementary Table 1), compared with individuals who never smoked in both childhood and adulthood, individuals who smoked in both childhood and adulthood (fully adjusted HR [95% CI]: 2.27 [2.19–2.35]) had the highest risk of all-cause mortality, followed by those who never smoked in childhood but smoked in adulthood (HR [95% CI]: 1.92 [1.85–2.00]), and those who smoked in childhood but had cessation in adulthood (HR [95% CI]: 1.33 [1.29–1.37]). Similarly, the HRs (95% CIs) of cancer-specific deaths were 3.33 (3.10–3.58), 2.68 (2.49–2.87) and 1.69 (1.59–1.80), respectively, and the HRs (95% CIs) of CVD-specific deaths were 2.50 (2.31–2.71), 2.21 (2.03–2.40), and 1.20 (1.13–1.29), respectively. Noteworthy, risk of mortality from chronic lower respiratory tract diseases was much higher in all three smoking groups, with the HRs (95% CIs) of 17.06 (14.51–20.06), 11.86 (10.20–13.81), and 7.45 (6.37–8.71), respectively (Supplementary Table 1).

### Association of duration since smoking cessation and age at smoking cessation among former smokers with all-cause and cause-specific mortality

Compared with never smokers, smokers with the shortest cessation duration had the highest risk of all-cause mortality, and those with the longest cessation duration had the lowest risk. The fully adjusted HRs (95% CIs) of all-cause mortality were 1.81 (1.73–1.89), 1.65 (1.56–1.75), 1.36 (1.30–1.41), 1.16 (1.11–1.21), and 1.08 (1.04–1.12), respectively, for those with cessation durations of <5, 5–9, 10–19, 20–29, and ≥30 years (Supplementary Table 3; Supplementary Figure 2). Similar patterns were found for cancer-specific, CVD-specific and chronic lower respiratory tract disease-specific mortality (Supplementary Table 3; Supplementary Figure 2).

**Figure 2:**
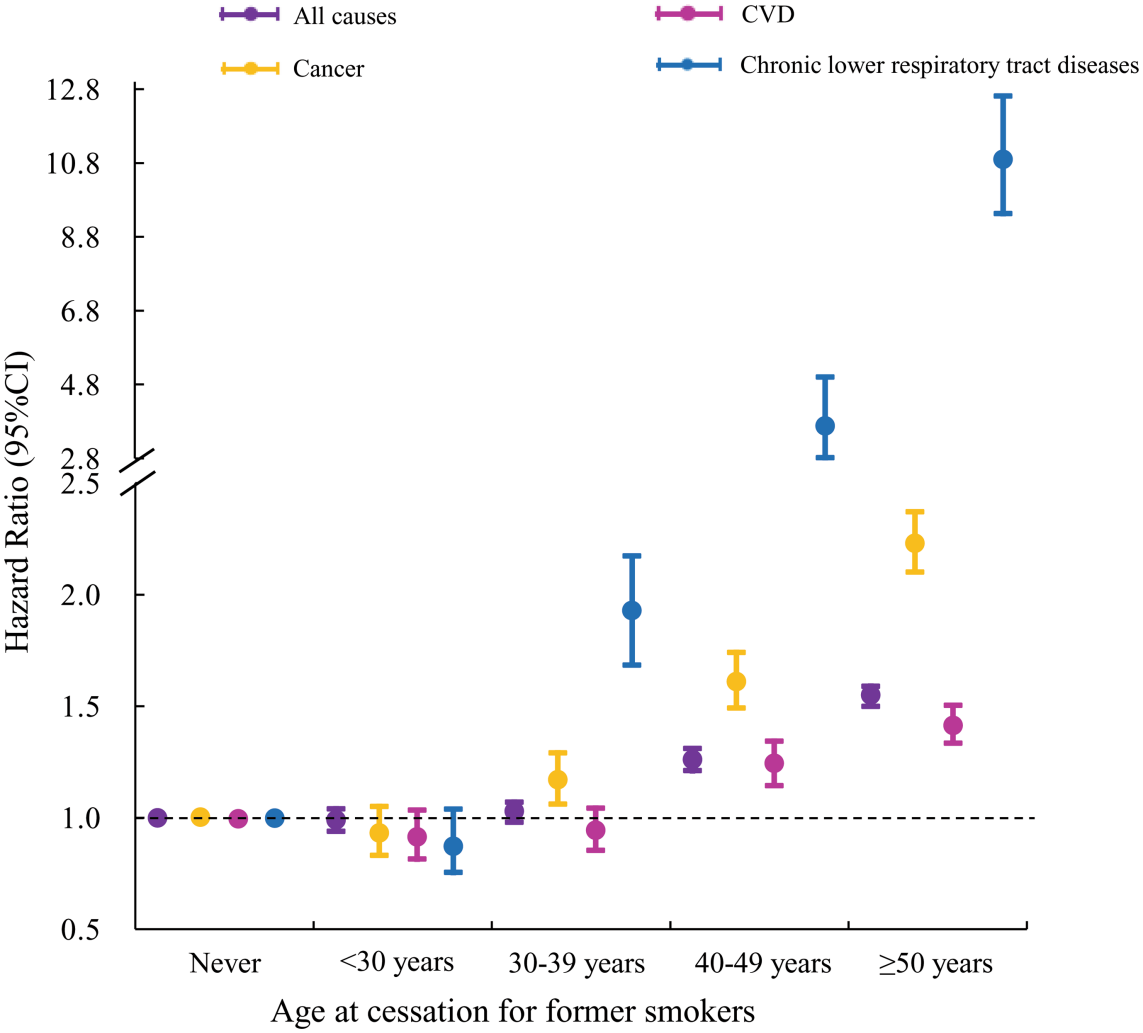
Association between age at smoking cessation for former smokers and all-cause and cause-specific mortality. Model was adjusted for age, gender, race/ethnicity, education, marital status, body mass index, alcohol intake, physical activity, and chronic conditions.

Comparing with never smokers, the fully adjusted HRs (95% CIs) of all-cause mortality were 0.99 (0.94–1.04), 1.03 (0.98–1.07), 1.26 (1.21–1.31), and 1.55 (1.50–1.59), respectively, for former smokers who stopped smoking at ages <30, 30–39, 40–49, and ≥50 years (Table 2; Figure 2). The corresponding HRs (95% CIs) of cancer-specific mortality were 0.93 (0.83–1.05), 1.17 (1.06–1.29), 1.61 (1.49–1.74), and 2.23 (2.10–2.37), respectively. The corresponding HRs (95% CIs) of CVD-specific mortality were 0.92 (0.82–1.04), 0.95 (0.86–1.05), 1.25 (1.15–1.35), and 1.42 (1.34–1.51), respectively. The corresponding HRs (95% CIs) of mortality from chronic lower respiratory tract diseases were 0.83 (0.56–1.23), 1.95 (1.47–2.59), 3.68 (2.98–4.54), and 10.90 (9.43–12.61), respectively (Table 2; Figure 2). These findings indicate that quitting smoking before 30 years was not significantly associated with an increased risk of mortality, especially for mortality from cancer and chronic lower respiratory tract diseases.

**Table 2:** Association between age at smoking cessation for former smokers and all-cause and cause-specific mortality.

Outcome	Never	Age at cessation for former smokers, years			
		<30	30–39	40–49	≥50
*N*	271,884	30,892	24,679	20,000	25,518
**All causes**					
No of deaths:	25,784	2,032	2,890	4,132	10,414
Model 1	1.00	0.89 (0.84–0.94)	0.96 (0.92–1.00)	1.21 (1.17–1.26)	1.60 (1.56–1.65)
Model 2	1.00	1.02 (0.97–1.07)	1.09 (1.04–1.13)	1.34 (1.29–1.40)	1.68 (1.64–1.73)
Model 3	1.00	0.99 (0.94–1.04)	1.03 (0.98–1.07)	1.26 (1.21–1.31)	1.55 (1.50–1.59)
**Cancer**					
No of deaths:	5,248	473	727	1,091	2,559
Model 1	1.00	0.90 (0.80–1.00)	1.14 (1.04–1.25)	1.59 (1.47–1.72)	2.33 (2.20–2.47)
Model 2	1.00	0.98 (0.88–1.10)	1.24 (1.13–1.36)	1.69 (1.56–1.83)	2.40 (2.27–2.55)
Model 3	1.00	0.93 (0.83–1.05)	1.17 (1.06–1.29)	1.61 (1.49–1.74)	2.23 (2.10–2.37)
**CVD**					
No of deaths:	6,136	421	651	970	2,394
Model 1	1.00	0.81 (0.72–0.91)	0.89 (0.81–0.98)	1.18 (1.09–1.28)	1.51 (1.42–1.60)
Model 2	1.00	0.95 (0.85–1.07)	1.03 (0.94–1.14)	1.35 (1.24–1.46)	1.62 (1.52–1.72)
Model 3	1.00	0.92 (0.82–1.04)	0.95 (0.86–1.05)	1.25 (1.15–1.35)	1.42 (1.34–1.51)
**Chronic lower respiratory tract diseases**					
No of deaths:	494	33	84	177	1,187
Model 1	1.00	0.68 (0.46–1.02)	1.64 (1.24–2.16)	3.22 (2.62–3.96)	10.61 (9.26–12.15)
Model 2	1.00	0.86 (0.58–1.28)	2.05 (1.54–2.72)	3.92 (3.16–4.85)	11.78 (10.17–13.64)
Model 3	1.00	0.83 (0.56–1.23)	1.95 (1.47–2.59)	3.68 (2.98–4.54)	10.90 (9.43–12.61)

Results are expressed as hazard ratios and 95% confidence intervals. Model 1: Adjusted for sex, age, and race/ethnicity. Model 2: Model 1 + education, marital status, body mass index, alcohol intake, and physical activity. Model 3: Model 2 + chronic conditions.

### Sensitivity analyses

Two sensitivity analyses were conducted to confirm the findings. First, the exclusion of those who died within the first 2 years of follow up showed similar results (Supplementary Tables 4–7). Second, the exclusion of those with any chronic conditions also had a little effect on the risk estimates (Supplementary Tables 4–7). Overall, these analyses showed similar findings as described above.

## Discussion

Based on nationally representative NHIS data, we found that all-cause and cause-specific (cancer, CVD and chronic lower respiratory tract diseases) mortality was directly associated with an early age at smoking initiation and indirectly with duration of smoking cessation. In particular, the mortality risk for those who smoked in childhood but had quit in adulthood was substantially reduced, yet still increased (*vs* never smokers), highlighting the long-term hazard of smoking in childhood on mortality in adulthood. This smoking-attributable mortality risk seemed largely reduced for former smokers who quit smoking before the age of 30 years.

### Comparison with other studies

To our knowledge, only limited studies have examined the association of cigarette smoking in childhood with all-cause mortality in adulthood. Evidence from the China Kadoorie Biobank showed that, compared with never smokers, smokers who began smoking before the age of 20 years had about 2-fold higher risk of all-cause mortality, but this study did not assess age at initiation in detail (5). A prospective cohort study in Cuba highlighted that early smoking initiation increased mortality risk in adulthood (e.g., 5–9 years vs. 15–19 years) (6). Our findings are consistent with this Cuban study. It is well-known that childhood is a crucial period for the adoption of often life-long behaviors and for organ growth and development. Smoking exposure starting early can cause DNA methylation and growth disturbances, leading the increased smoking-related morbidity and mortality (15, 16). However, early smoking initiation does not necessarily equate to a longer exposure to smoking across the lifetime. A substantial proportion of young smokers quit smoking in adulthood and the health risk of quitters can be mitigated.

To our knowledge, our study is the first to assess the effects of smoking status changes from childhood to adulthood on the all-cause and cause-specific mortality in adulthood. Our findings show that, compared with never smokers from childhood to adulthood (never smokers group), individuals who smokers during both childhood and adulthood (persistent smokers group) had the highest risk of mortality, followed by those who did not smoke in childhood but smoked in adulthood (incident smokers group). It is notable that the risk for individuals who smoked in childhood but quit smoking in adulthood (cessation group) was largely reduced compared with individuals who smoked in both childhood and adulthood or individuals who started to smoke in adulthood, highlighting the benefit of cessation in adulthood. However, mortality risk was still increased in quitters, highlighting the importance of never smoking across the lifetime.

The Cuban study showed that smoking cessation before the age of about 40 years could eliminate almost all the excess smoking-related all-cause mortality (6). However, the Cuban study did not assess the effect of early vs. later smoking cessation on the risk of cause-specific mortality because of the limited cases of deaths in their study. In our study with a large number of individuals, we found that former smokers who had quit smoking before the age of 30 years had a cause-specific mortality risk (cancer, CVD and chronic lower respiratory tract diseases) that was not substantially increased compared with never smokers. For smokers who had quit smoking after the age of 30 years, the adverse impact of cigarette smoking increased with each decade of delayed cessation, highlighting the importance of early quitting (17–20). These findings reinforce the importance of smoking cessation campaigns and programs.

### Strengths and limitations of this study

Our study has several strengths. First, this study used a large participants sample (*N* = 472,887) form a nationally representative survey to investigate the association between cigarette smoking in childhood and mortality in adulthood assessed by linkage with mortality data during a substantial follow-up after collecting the survey data. Second, many potential confounders including sociodemographic factors, lifestyle variables and chronic conditions were adjusted in the regression models to reduce biases due to potential confounding variables. In addition, several sensitivity analyses confirmed the stability of our findings. Finally, to our knowledge, our study was the first to investigate the association of changes in cigarette smoking status from childhood to adulthood with all-cause and cause-specific mortality in adulthood. However, our study also has several limitations. Firstly, this study used self-reported data on cigarette smoking. However, self-reported information on current smoking has been shown to strongly correlate with serum cotinine levels (21). Information gathered in adults about previous smoking habits in childhood may also be quite reliable as smoking in childhood often is a memorable behavior for individuals. Secondly, although many potential confounders were adjusted in the regression models, the possibility of residual or unmeasured confounding could not be completely ruled out. Last, using self-reported co-morbidities in this study may have introduced inaccuracies due to potential recall bias and underestimates.

## Conclusion

Our findings indicated that cigarette smoking in childhood significantly increased the risk of all-cause and cause-specific mortality in adulthood and lifetime non-smoking history was associated with the lowest risk of mortality. Smoking cessation before the age of 30 years appeared to largely reduce the mortality risk due to former smoking, and smoking cessation at later ages also showed health benefits although at a lower scale. Our findings highlight the benefits on health of never smoking and, for smokers, of early smoking cessation. More generally, our findings also support the need for comprehensive public health policy and programs to reduce smoking demand and prevent cigarette smoking, particularly in childhood, along the measures advocated in the Framework Convention of Tobacco Control.

## Data availability statement

The original contributions presented in the study are included in the article/Supplementary material, further inquiries can be directed to the corresponding author.

## Ethics statement

The studies involving human participants were reviewed and approved by the NHIS anonymized data are publicly available and ethical review by the corresponding author’s Ethics Committee is not requested. The patients/participants provided their written informed consent to participate in this study.

## Author contributions

XL and PB conceptualized and designed the study, drafted the initial manuscript, and reviewed and revised the manuscript. JS and MZ designed the data collection instruments, collected data, carried out the initial analyses, and reviewed and revised the manuscript. BX conceptualized and designed the study, coordinated and supervised data collection, and critically reviewed the manuscript for important intellectual content. All authors contributed to the article and approved the submitted version.

## Funding

This work was supported by the Youth Team of Humanistic and Social Science (20820IFYT1902). The funder had no role in the design and conduct of the study; collection, management, analysis, and interpretation of the data; preparation, review, or approval of the article; and decision to submit the article for publication.

## Conflict of interest

The authors declare that the research was conducted in the absence of any commercial or financial relationships that could be construed as a potential conflict of interest.

## Publisher’s note

All claims expressed in this article are solely those of the authors and do not necessarily represent those of their affiliated organizations, or those of the publisher, the editors and the reviewers. Any product that may be evaluated in this article, or claim that may be made by its manufacturer, is not guaranteed or endorsed by the publisher.
