# Supplementary material for: Cigarette smoking in childhood and risk of all-cause and cause-specific mortality in adulthood
Source: Front Public Health. 2023 Jul 6;11:1051597. doi: 10.3389/fpubh.2023.1051597 (PMC10359425; doi:10.3389/fpubh.2023.1051597)
Supplement: Supplementary file 1 [file Data_Sheet_1.pdf]

Supplementary Materials

Supplementary Table 1. Baseline characteristics of the participants to NHIS in 1997-2014 according to age at smoking initiation

Characteristics	Never	Age at smoking initiation for participants who smoked during childhood but had quit smoking during adulthood					Age at smoking initiation for participants who smoked during childhood and were currently smoking during adulthood				
		Any age	6-9	10-14	15-17	≥18	Any age	6-9	10-14	15-17	≥18
N	271884	101089	1460	16024	34005	49600	99914	1650	17979	33907	46378
**Age, yrs, %**											
18-39	45.6	20.3	10.9	23.1	23.5	17.3	46.0	36.5	48.0	51.1	41.5
40-59	34.1	38.7	32.7	39.2	39.8	38.0	40.8	42.1	40.2	37.9	43.4
≥60	20.2	41.0	56.4	37.7	36.8	44.8	13.2	21.4	11.8	11.0	15.1
**Sex, %**											
Men	44.1	57.3	84.9	65.3	58.9	52.7	54.4	70.9	57.3	54.9	52.2
Women	55.9	42.7	15.1	34.7	41.1	47.3	45.6	29.1	42.7	45.1	47.8
**Race/ethnicity, %**											
White	65.0	81.3	82.5	82.0	83.1	79.8	75.5	78.8	80.4	78.5	71.0
Black	13.1	8.3	6.0	6.3	6.5	8.3	9.0	8.3	8.2	10.1	14.9
Hispanic	15.6	7.3	9.7	9.3	8.1	8.0	11.9	8.4	8.8	8.4	9.7
Other	6.3	3.1	1.8	2.4	2.3	3.9	3.6	4.5	2.6	3.0	4.4
**Education, %**											
<High school	14.4	15.3	40.7	22.0	16.3	11.6	20.9	42.9	29.2	22.9	15.1
High school	25.0	28.9	28.3	31.7	30.9	26.4	36.9	33.8	37.3	39.4	34.9
>High school	60.6	55.8	30.9	46.3	52.8	62.0	42.2	23.3	33.5	37.7	50.0

Marital status, %											
Married	56.4	65.7	64.5	65.6	67.0	64.9	45.6	46.1	44.5	45.1	46.4
Divorced/separated/widowed	14.8	20.5	24.1	19.1	18.6	22.3	20.5	24.9	20.4	18.5	21.9
Never married	28.8	13.7	11.4	15.3	14.4	12.8	33.9	29.0	35.1	36.4	31.7
**BMI category, kg/m^2, %**											
<25.0	40.6	31.9	23.8	28.1	31.8	33.4	43.8	38.8	43.2	45.5	42.9
25.0-29.9	34.4	38.8	38.8	38.2	39.4	38.6	33.4	33.4	32.2	32.7	34.3
≥30.0	25.0	29.3	37.4	33.7	28.8	28.0	22.9	27.8	24.6	21.8	22.9
**Drinking, %**											
Lifetime abstainer	31.4	8.3	10.9	7.5	7.8	8.9	9.4	8.9	8.5	9.2	10.0
Former drinker	11.1	23.1	41.6	28.0	22.2	21.5	15.4	24.7	18.5	14.5	14.5
Light to moderate	54.9	62.7	43.2	57.7	63.9	64.1	64.0	51.1	58.8	64.7	66.0
Heavy	2.6	5.9	4.3	6.8	6.1	5.5	11.2	15.3	14.2	11.6	9.5
**Physical activity (meeting recommendations), %**											
No	53.6	54.5	66.5	56.9	54.7	53.1	61.1	70.1	66.2	61.6	58.3
Yes	46.4	45.5	33.5	43.1	45.3	46.9	38.9	29.9	33.8	38.4	41.7
**Physician-diagnosed diseases, %**											
Hypertension	24.0	38.7	49.8	38.9	36.5	39.9	23.6	33.7	24.7	21.5	24.5
Heart disease	9.1	19.6	34.7	22.5	18.3	19.2	10.7	25.8	13.4	9.8	9.7
Stroke	1.9	4.3	8.5	5.1	3.9	4.3	2.5	6.0	3.3	2.2	2.2
Diabetes	6.6	12.0	24.0	14.3	10.9	11.7	6.0	11.0	7.1	5.4	5.9
Cancer	6.1	13.1	19.3	12.6	12.4	13.6	6.1	10.1	7.5	5.5	6.0

Supplementary Table 2. Association between cigarette smoking status in childhood (age 6-17 years) and adulthood (age 18-85 years) and all-cause and cause-specific mortality in adulthood

Outcome	Smoking status in childhood and adulthood			
	Never (in both childhood and adulthood)	Smoking in childhood but cessation in adulthood	Never in childhood but smoking in adulthood	Smoking in childhood and in adulthood
**N**	271884	49849	46378	53536
**All causes**				
No of deaths:	25784	9426	6075	7174
Model 1	1.00	1.38 (1.34-1.42)	1.97 (1.90-2.04)	2.60 (2.51-2.69)
Model 2	1.00	1.44 (1.40-1.49)	1.95 (1.88-2.03)	2.39 (2.30-2.47)
Model 3	1.00	1.33 (1.29-1.37)	1.92 (1.85-2.00)	2.27 (2.19-2.35)
**Cancer**				
No of deaths:	5248	2529	1770	2223
Model 1	1.00	1.80 (1.70-1.91)	2.73 (2.55-2.93)	3.76 (3.51-4.02)
Model 2	1.00	1.84 (1.73-1.95)	2.71 (2.53-2.91)	3.51 (3.27-3.76)
Model 3	1.00	1.69 (1.59-1.80)	2.68 (2.49-2.87)	3.33 (3.10-3.58)
**CVD**				
No of deaths:	6136	2076	1247	1379
Model 1	1.00	1.28 (1.20-1.36)	2.29 (2.13-2.46)	2.98 (2.76-3.22)
Model 2	1.00	1.35 (1.27-1.44)	2.29 (2.12-2.47)	2.72 (2.51-2.94)
Model 3	1.00	1.20 (1.13-1.29)	2.21 (2.03-2.40)	2.50 (2.31-2.71)
**Chronic lower respiratory tract diseases**				
No of deaths:	494	784	527	628

Model 1	1.00	7.46 (6.42-8.66)	13.38 (11.56-15.48)	22.08 (18.93-25.76)
Model 2	1.00	8.11 (6.93-9.48)	12.16 (10.48-14.11)	18.04 (15.35-21.19)
Model 3	1.00	7.45 (6.37-8.71)	11.86 (10.20-13.81)	17.06 (14.51-20.06)

Results are expressed as hazard ratios and 95% confidence intervals.

Model 1: Adjusted for sex, age, and race/ethnicity.

Model 2: Model 1+education, marital status, body mass index, alcohol intake, and physical activity.

Model 3: Model 2+chronic conditions.

Supplementary Table 3. Association between cessation duration for former smokers and risk of all-cause and cause-specific mortality

Outcome	Never	Duration since smoking cessation, years				
		<5	5-9	10-19	20-29	≥30
**N**	271884	22767	13422	23872	19426	21602
**All causes**						
No of deaths:	25784	2921	2114	4433	4010	5990
Model 1	1.00	1.94 (1.87-2.03)	1.75 (1.65-1.84)	1.35 (1.30-1.41)	1.12 (1.07-1.16)	1.01 (0.98-1.04)
Model 2	1.00	1.95 (1.87-2.04)	1.80 (1.70-1.90)	1.45 (1.40-1.51)	1.24 (1.19-1.29)	1.15 (1.11-1.18)
Model 3	1.00	1.81 (1.73-1.89)	1.65 (1.56-1.75)	1.36 (1.30-1.41)	1.16 (1.11-1.21)	1.08 (1.04-1.12)
**Cancer**						
No of deaths:	5248	890	620	1131	998	1211
Model 1	1.00	2.68 (2.48-2.91)	2.44 (2.21-2.70)	1.72 (1.59-1.85)	1.37 (1.27-1.49)	1.10 (1.02-1.18)
Model 2	1.00	2.69 (2.48-2.91)	2.49 (2.25-2.75)	1.80 (1.66-1.94)	1.48 (1.37-1.61)	1.20 (1.11-1.30)
Model 3	1.00	2.55 (2.34-2.77)	2.33 (2.10-2.58)	1.71 (1.58-1.85)	1.39 (1.28-1.52)	1.10 (1.02-1.19)
**CVD**						
No of deaths:	6136	556	437	1009	928	1506
Model 1	1.00	2.08 (1.89-2.30)	1.86 (1.67-2.08)	1.46 (1.35-1.58)	1.08 (0.99-1.18)	0.92 (0.86-0.98)
Model 2	1.00	2.08 (1.88-2.30)	1.90 (1.71-2.13)	1.58 (1.46-1.71)	1.22 (1.12-1.34)	1.07 (1.00-1.15)
Model 3	1.00	1.82 (1.65-2.01)	1.66 (1.48-1.85)	1.40 (1.29-1.52)	1.10 (1.01-1.21)	1.01 (0.94-1.08)
**Chronic lower respiratory tract diseases**						
No of deaths:	494	352	249	390	250	240
Model 1	1.00	20.63 (17.40-24.47)	15.29 (12.59-18.56)	8.10 (6.76-9.70)	4.17 (3.44-5.07)	1.96 (1.62-2.36)
Model 2	1.00	20.46 (17.14-24.42)	15.97 (13.01-19.61)	9.13 (7.57-11.02)	4.98 (4.07-6.09)	2.41 (1.98-2.93)
Model 3	1.00	18.79 (15.71-22.47)	14.83 (12.09-18.19)	8.48 (7.03-10.22)	4.64 (3.79-5.68)	2.26 (1.86-2.75)

Results are expressed as hazard ratios and 95% confidence intervals.

Model 1: Adjusted for sex, age, and race/ethnicity.

Model 2: Model 1+education, marital status, body mass index, alcohol intake, and physical activity.

Model 3: Model 2+chronic conditions.

Supplementary Table 4. Sensitivity analysis of the association between age at smoking initiation and all-cause and cause-specific mortality

Outcome	Never	Age at smoking initiation for participants who smoked during childhood but had quit smoking during adulthood				Age at smoking initiation for participants who smoked during childhood and were currently smoking during adulthood			
		6-9	10-14	15-17	≥18	6-9	10-14	15-17	≥18
Exclusion of those who died within the first 2 years of follow up †									
All causes	1.00	1.41 (1.22-1.63)	1.39 (1.32-1.46)	1.29 (1.24-1.34)	1.22 (1.19-1.26)	2.59 (2.25-2.97)	2.53 (2.40-2.68)	2.29 (2.18-2.39)	1.98 (1.91-2.07)
Cancer	1.00	1.91 (1.49-2.46)	1.78 (1.62-1.97)	1.60 (1.48-1.72)	1.37 (1.28-1.47)	3.88 (3.06-4.91)	3.56 (3.19-3.96)	3.29 (3.01-3.59)	2.75 (2.56-2.96)
CVD	1.00	1.01 (0.71-1.45)	1.26 (1.13-1.40)	1.16 (1.06-1.26)	1.20 (1.12-1.27)	3.40 (2.57-4.51)	2.71 (2.39-3.08)	2.40 (2.16-2.66)	2.26 (2.08-2.47)
Chronic lower respiratory tract diseases	1.00	9.27 (5.75-14.95)	9.08 (7.40-11.13)	6.51 (5.38-7.87)	5.50 (4.71-6.42)	19.14 (12.19-30.07)	22.27 (17.99-27.56)	15.16 (12.52-18.36)	12.64 (10.76-14.84)
Exclusion of those with any chronic conditions ‡									
All causes	1.00	1.32 (1.02-1.70)	1.26 (1.14-1.40)	1.24 (1.15-1.34)	1.18 (1.12-1.25)	2.61 (2.13-3.20)	2.17 (1.99-2.36)	2.07 (1.93-2.22)	1.84 (1.73-1.96)
Cancer	1.00	2.13 (1.70-2.68)	2.00 (1.83-2.19)	1.74 (1.63-1.86)	1.53 (1.44-1.63)	4.16 (3.36-5.15)	3.79 (3.44-4.19)	3.28 (3.02-3.55)	2.70 (2.52-2.89)
CVD	1.00	1.50 (1.12-2.00)	1.53 (1.40-1.68)	1.28 (1.19-1.38)	1.34 (1.26-1.42)	3.92 (3.06-5.02)	2.88 (2.55-3.25)	2.61 (2.37-2.88)	2.32 (2.15-2.51)
Chronic lower respiratory tract diseases	1.00	10.63 (7.00-16.15)	9.94 (8.29-11.92)	7.29 (6.11-8.69)	5.94 (5.15-6.85)	20.34 (13.44-30.76)	23.55 (19.41-28.59)	15.75 (13.24-18.73)	12.27 (10.60-14.21)

Results are expressed as hazard ratios and 95% confidence intervals.

† Adjusted for sex, age, race/ethnicity, education, marital status, body mass index, alcohol intake, physical activity and chronic conditions.

‡ Adjusted for sex, age, race/ethnicity, education, marital status, body mass index, alcohol intake, and physical activity.

Supplementary Table 5. Sensitivity analysis of the association between cigarette smoking status in childhood (age 6-17 years) and adulthood (age 18-85 years) and all-cause and cause-specific mortality in adulthood

Outcome	Smoking status in childhood and adulthood			
	Never (in both childhood and adulthood)	Smoking in childhood but cessation in adulthood	Never in childhood but smoking in adulthood	Smoking in childhood and in adulthood
Exclusion of those who died within the first 2 years of follow up †				
All causes	1.00	1.32 (1.28-1.37)	1.97 (1.89-2.05)	2.35 (2.26-2.44)
Cancer	1.00	1.67 (1.56-1.79)	2.77 (2.57-2.98)	3.43 (3.18-3.71)
CVD	1.00	1.18 (1.10-1.26)	2.23 (2.05-2.44)	2.51 (2.31-2.73)
Chronic lower respiratory tract diseases	1.00	7.28 (6.13-8.65)	12.40 (10.54-14.59)	17.10 (14.35-20.38)
Exclusion of those with any chronic conditions ‡				
All causes	1.00	1.44 (1.40-1.49)	1.95 (1.88-2.03)	2.39 (2.30-2.47)
Cancer	1.00	1.84 (1.73-1.95)	2.71 (2.53-2.91)	3.51 (3.27-3.76)
CVD	1.00	1.35 (1.27-1.44)	2.29 (2.12-2.47)	2.72 (2.51-2.94)
Chronic lower respiratory tract diseases	1.00	8.11 (6.93-9.48)	12.16 (10.48-14.11)	18.04 (15.35-21.19)

Results are expressed as hazard ratios and 95% confidence intervals.

† Adjusted for sex, age, race/ethnicity, education, marital status, body mass index, alcohol intake, physical activity and chronic conditions.

‡ Adjusted for sex, age, race/ethnicity, education, marital status, body mass index, alcohol intake, and physical activity.

Supplementary Table 6. Sensitivity analysis of the association between cessation duration for former smokers and risk of all-cause and cause-specific mortality

Outcome	Never	Duration since smoking cessation, years				
		<5	5-9	10-19	20-29	30+
Exclusion of those who died within the first 2 years of follow up †						
All causes	1.00	1.74 (1.66-1.83)	1.62 (1.52-1.72)	1.33 (1.27-1.38)	1.15 (1.10-1.20)	1.08 (1.04-1.12)
Cancer	1.00	2.34 (2.13-2.58)	2.17 (1.94-2.43)	1.61 (1.47-1.75)	1.36 (1.24-1.49)	1.10 (1.01-1.20)
CVD	1.00	1.74 (1.56-1.95)	1.64 (1.45-1.85)	1.32 (1.21-1.45)	1.09 (0.98-1.20)	0.99 (0.91-1.07)
Chronic lower respiratory tract diseases	1.00	17.03 (13.95-20.79)	14.73 (11.81-18.37)	8.17 (6.63-10.06)	4.59 (3.69-5.71)	2.22 (1.81-2.73)
Exclusion of those with any chronic conditions ‡						
All causes	1.00	1.95 (1.87-2.04)	1.80 (1.70-1.90)	1.45 (1.40-1.51)	1.24 (1.19-1.29)	1.15 (1.11-1.18)
Cancer	1.00	2.69 (2.48-2.91)	2.49 (2.25-2.75)	1.80 (1.66-1.94)	1.48 (1.37-1.61)	1.20 (1.11-1.30)
CVD	1.00	2.08 (1.88-2.30)	1.90 (1.71-2.13)	1.58 (1.46-1.71)	1.22 (1.12-1.34)	1.07 (1.00-1.15)
Chronic lower respiratory tract diseases	1.00	20.46 (17.14-24.42)	15.97 (13.01-19.61)	9.13 (7.57-11.02)	4.98 (4.07-6.09)	2.41 (1.98-2.93)

Results are expressed as hazard ratios and 95% confidence intervals.

† Adjusted for sex, age, race/ethnicity, education, marital status, body mass index, alcohol intake, physical activity and chronic conditions.

‡ Adjusted for sex, age, race/ethnicity, education, marital status, body mass index, alcohol intake, and physical activity.

Supplementary table 7. Sensitivity analysis of the association between age at smoking cessation for former smoker and all-cause and cause-specific mortality

Outcome	Never	Age at cessation for former smoker, years			
		<30	30-39	40-49	≥50
Exclusion of those who died within the first 2 years of follow up †					
All causes	1.00	0.98 (0.92-1.03)	1.02 (0.97-1.07)	1.25 (1.19-1.30)	1.52 (1.47-1.57)
Cancer	1.00	0.91 (0.81-1.03)	1.14 (1.02-1.26)	1.57 (1.44-1.72)	2.13 (1.98-2.28)
CVD	1.00	0.92 (0.81-1.04)	0.95 (0.85-1.06)	1.20 (1.10-1.32)	1.38 (1.29-1.47)
Chronic lower respiratory tract diseases	1.00	0.83 (0.54-1.27)	1.95 (1.46-2.60)	3.66 (2.92-4.58)	10.61 (9.07-12.42)
Exclusion of those with any chronic conditions ‡					
All causes	1.00	1.02 (0.97-1.07)	1.09 (1.04-1.13)	1.34 (1.29-1.40)	1.68 (1.64-1.73)
Cancer	1.00	0.98 (0.88-1.10)	1.24 (1.13-1.36)	1.69 (1.56-1.83)	2.40 (2.27-2.55)
CVD	1.00	0.95 (0.85-1.07)	1.03 (0.94-1.14)	1.35 (1.24-1.46)	1.62 (1.52-1.72)
Chronic lower respiratory tract diseases	1.00	0.86 (0.58-1.28)	2.05 (1.54-2.72)	3.92 (3.16-4.85)	11.78 (10.17-13.64)

Results are expressed as hazard ratios and 95% confidence intervals.

† Adjusted for sex, age, race/ethnicity, education, marital status, body mass index, alcohol intake, physical activity and chronic conditions.

‡ Adjusted for sex, age, race/ethnicity, education, marital status, body mass index, alcohol intake, and physical activity.

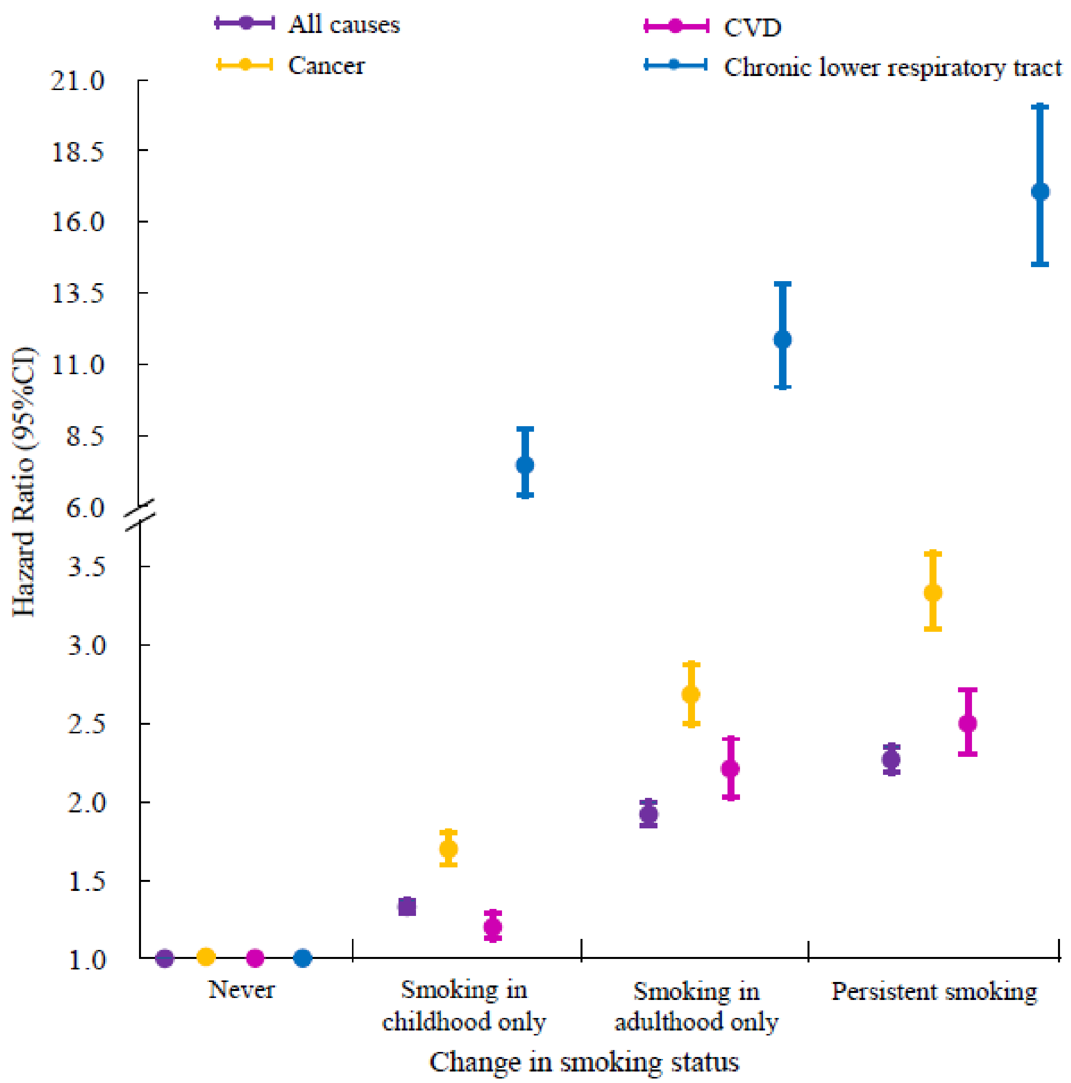

Supplementary Figure 1. Associations of cigarette smoking status in childhood (age 6-17 years) and adulthood (age 18-85 years) with all-cause and cause-specific mortality in adulthood. *Model was adjusted for age, gender, race/ethnicity, education, marital status, body mass index, alcohol intake, physical activity, and chronic conditions.*

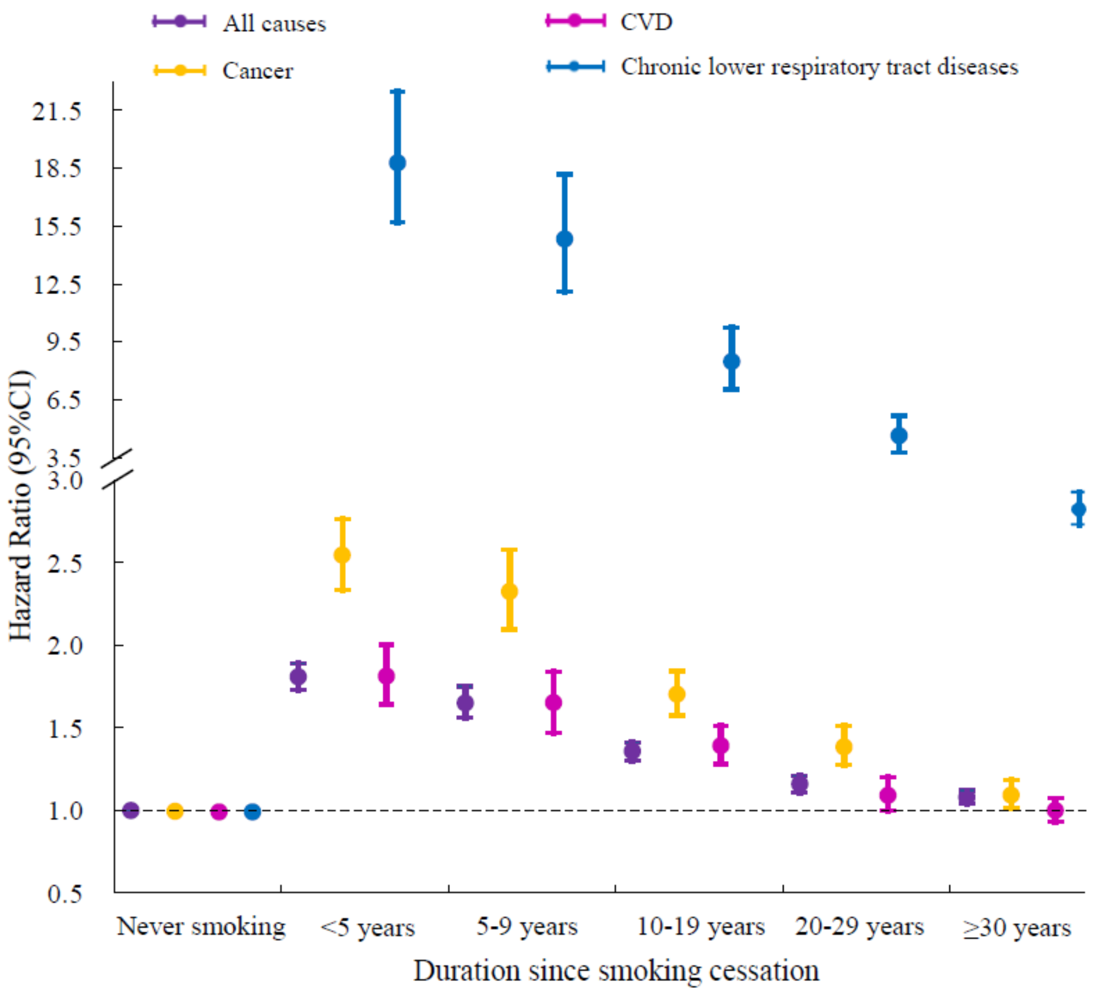

Supplementary Figure 2. Associations of cessation duration for former smokers with risk of all-cause and cause-specific mortality. *Model was adjusted for age, gender, race/ethnicity, education, marital status, body mass index, alcohol intake, physical activity, and chronic conditions.*
